# Certified causes of death in patients with mesothelioma in South East England

**DOI:** 10.1186/1471-2407-9-28

**Published:** 2009-01-23

**Authors:** Catherine Okello, Tom Treasure, Andrew G Nicholson, Julian Peto, Henrik Møller

**Affiliations:** 1 King's College London, Thames Cancer Registry, 42 Weston Street, London, SE1 3QD, UK; 2 Clinical Operational Research Unit, University College London, London UK; 3 Royal Brompton Hospital, Department of Histopathology, London, UK; 4 London School of Hygiene and Tropical Medicine, London, UK

## Abstract

**Background:**

Mesothelioma is a highly fatal cancer that is caused by exposure to asbestos fibres. In many populations, the occurrence of mesothelioma is monitored with the use of mortality data from death certification. We examine certified causes of death of patients who have been diagnosed with mesothelioma, and assess the validity of death certification data as a proxy for mesothelioma incidence.

**Methods:**

We extracted mesothelioma registrations in the South East of England area between 2000 and 2004 from the Thames Cancer Registry database. We retained for analysis 2200 patients who had died at the time of analysis, after having excluded seven dead cases where the causes of death were not known to the cancer registry. The 2200 deaths were classified hierarchically to identify (1) mesothelioma deaths, (2) deaths certified as lung cancer deaths or (3) deaths from unspecified cancer, and (4) deaths from other causes.

**Results:**

87% of the patients had mesothelioma mentioned on the death certificate. 6% had no mention of mesothelioma but included lung cancer as a cause of death. Another 6% had no mention of mesothelioma or lung cancer, but included an unspecified cancer as a cause of death. Lastly, 2% had other causes of death specified on the death certificate.

**Conclusion:**

This analysis suggests that official mortality data may underestimate the true occurrence of mesothelioma by around 10%.

## Background

Mesothelioma is a rare form of cancer that arises from the pleura or, less often, from the peritoneum. The disease is almost always fatal and the median time between diagnosis and death is less than one year [1,2]. Mesothelioma is strongly associated with occupational exposure to asbestos fibres. Industries with high exposure to asbestos in the past included mining, shipyard working and asbestos cement manufacture.

The annual numbers of mesothelioma deaths in Great Britain increased 12-fold in the period from 1968 to 2001[3] and an epidemiological model that incorporated the historical use of asbestos suggested that the annual numbers of deaths will continue to rise to a peak at around 1950–2400 deaths per year in 2011–2015.

The aim of this analysis was to examine the certified causes of death of patients diagnosed with mesothelioma, and assess the validity of mortality data as a proxy for data on mesothelioma incidence.

## Methods

Initially, we extracted the numbers of mesothelioma cancer registrations and mesothelioma deaths nationally for 2004 from the Cancer Information System (CIS), which provides cancer registration and cancer mortality data from the whole of England.

Thereafter, we extracted cancer registrations of patients diagnosed with mesothelioma in the South East of England area between 2000 and 2004 from the Thames Cancer Registry database. Patients who had cause of death specified on the death certificate were identified and used for the analysis.

The deaths were classified hierarchically as follows: Firstly, we identified death certificates that mentioned mesothelioma as one of the causes of death. Secondly, among the remaining death certificates, we identified those that mentioned lung cancer as a cause of death. Third, among the now remaining death certificates, we identified those that mentioned an unspecified cancer as a cause of death. Last, there remained a group of death certificates that included no mention of mesothelioma, lung cancer or unspecified cancer.

## Results

In 2004 there were 1847 cases of mesothelioma and 1629 deaths from mesothelioma in the whole of England. These numbers, which are based on nationwide cancer registration and official mortality statistics, respectively, suggest crudely that around 88% of mesothelioma cases die from the disease. An analysis of one area of England was therefore set up to explore this in more detail using individually linked information on mesothelioma incidence and the causes of death in these patients.

There were 2433 patients diagnosed with mesothelioma in South East England in the period 2000–2004. 1985 of the patients were males and 448 were females. 2207 of these patients had died at the time of analysis. Out of these, 2200 of the patients had their causes of death certified and these were identified and retained for analysis. The remaining seven patients for whom we did not have the causes of death were excluded from further analysis.

Table 1 shows that 87% of the mesothelioma patients had mesothelioma mentioned on the death certificate. 6% had no mention of mesothelioma but included lung cancer as a cause of death. Another 6% had no mention of mesothelioma or lung cancer, but included an unspecified cancer as a cause of death. Lastly, 2% had other causes of death specified on the death certificate.

**Table 1:** Analysis of certified causes of death in 2200 mesothelioma patients who had died.

Causes of death extracted from ICD version 10 coded causes of death in the death certificate record	N	%
		

Death certificate mentioned mesothelioma.	1905	87

No mention of mesothelioma. Death certificate mentioned lung cancer.	128	6

No mention of mesothelioma or lung cancer. Death certificate mentioned an unspecified cancer.	123	6

All other	44	2

Total	2200	100

The proportion of death certificates that mentioned mesothelioma was dependent on age at diagnosis. It was 90% in the age-group up to 69 years, 86% in the 70–79 years age-group, and 78% in patients 80 years and older. Correspondingly, the apparent misclassification as lung cancer deaths (6% overall) and as unspecified cancer deaths (6% overall) were highest in the 80+ years age-group at 11% and 7%, respectively.

## Discussion

Overall, most patients diagnosed with mesothelioma had mesothelioma recorded on the death certificate. However, about 12% of mesothelioma patients were certified as dying from lung cancer or from an unspecified cancer and this proportion increased with age.

Since the introduction of the 10^th ^version if the International Classification of Diseases (ICD), mesothelioma has had a unique code (C45) which enables more complete registration of mesothelioma as a cause of death. In the previous revisions of ICD, mesothelioma deaths were commonly assigned to code 163 (Malignant neoplasm of pleura), 162 (Malignant neoplasm of bronchus and lung) or 199 (Malignant neoplasm, unspecified site) [4].

A previous study from Scotland described the singular, underlying cause of death in mesothelioma patients before and after the introduction of ICD version 10 in year 2000 [4]. The specificity of the coding was greatly improved from 40% of cases coded to 163 (Malignant neoplasm of pleura) in ICD version 9 to 74% of cases coded to a mesothelioma code (C45) in ICD version 10. The present study extends the Scottish study of the ICD version 10 coded underlying cause of death in 607 mesothelioma cases to 2200 mesothelioma cases and extends the analysis to all the causes of death that are listed on the death certificate.

Record linkage analysis, similar to the present study, has been carried out for a variety of diseases, e.g. [5,6]. Compared with many other diseases, the certification rate for mesothelioma is relatively high. In the main, this is a consequence of the poor prognosis and high fatality of mesothelioma.

We have not verified the mesothelioma cancer registrations for the purpose of this study. It is possible that a proportion of the mesothelioma cases were misclassified lung cancers or metastases from other cancers. Such misclassification is, however, likely to be small, and could only account for a small part of the deaths coded as lung cancer deaths or deaths from unspecified cancers. This is because, in the vast majority of patients with suspected malignancy involving the pleura, most will now have biopsy confirmation and a recent validation study in relation to the MESO-1 chemotherapy trial has shown a very high incidence of correct diagnosis pre-mortem for mesothelioma [7], due at least in part to the relatively recent availability commercially of antibodies that show good specificity and sensitivity for a mesothelial phenotype [8]. The likelihood of people being managed without biopsy is further decreased by the need for tissue diagnosis in relation to any potential medicolegal consequence with regards to occupational exposure to asbestos.

Mesothelioma patients may possibly die from any cause of death. The 2% of cases with "All other" causes of death included a variety of diseases and causes of death. The main concern is not about these relatively few patients, but the cases where the cause of death was attributed to lung cancer or to an unspecified cancer. It is most likely that many of these records erroneously omitted the mention of mesothelioma as a contributory cause of death.

## Conclusion

Incorrect death certification is a serious error, not least for deaths that are caused by occupational or environmental exposures. This analysis suggests that the official mortality data may underestimate the true occurrence of mesothelioma by around 10%.

## Competing interests

The authors declare that they have no competing interests.

## Authors' contributions

CO and HM designed the study and CO analysed the death certificates and wrote the first draft. HM edited subsequent drafts. All authors contributed to the interpretation of results and revised consecutive drafts for intellectual content.

## Pre-publication history

The pre-publication history for this paper can be accessed here:

http://www.biomedcentral.com/1471-2407/9/28/prepub
